# The ACE2/Ang-(1-7)/Mas Axis Regulates the Development of Pancreatic Endocrine Cells in Mouse Embryos

**DOI:** 10.1371/journal.pone.0128216

**Published:** 2015-06-01

**Authors:** Lin Wang, Juan Liang, Po Sing Leung

**Affiliations:** School of Biomedical Sciences, Faculty of Medicine, The Chinese University of Hong Kong, Hong Kong, China; Max-Delbrück Center for Molecular Medicine (MDC), GERMANY

## Abstract

Angiotensin-converting enzyme 2 (ACE2), its product Angiotensin-(1-7) [Ang-(1-7)], and Ang-(1-7) receptor Mas, have been shown to regulate organogenesis during embryonic development in various species. However, it is not known whether a local ACE2/Ang-(1-7)/Mas axis is present in the fetal pancreas. It is hypothesized that there is a local ACE2/Ang-(1-7)/Mas axis in the embryonic pancreas in mice that is involved in regulating islet cell development. To address this issue, the endogenous expression profile of axis constituents in embryonic mouse pancreata was examined. Involvement of the ACE2 axis in the regulation of pancreatic development was also examined. The present experiments showed in an *in vivo* animal model that endogenous expression levels of ACE2 and the Mas receptor were upregulated in mouse pancreata in late embryogenesis, peaking on embryonic day E16.5, when it reached 3 folds compared to that seen at E12.5. Consistently, endogenous expression of Ang-(1-7) also peaked at E16.5. Treatment with the ACE2 inhibitor DX600 did not alter islet development. However, prenatal treatment with A779, a Mas receptor antagonist, reduced the β-cell to α-cell ratio in neonatal islets, impaired islet insulin secretory function, and impaired the pups’ glucose tolerance. In *ex vivo* pancreas explant cultures, A779 again decreased the β-cell to α-cell ratio, apparently through its effects on β-cell proliferation (reduced proliferation shown with Ki67 staining), and also decreased *Insulin* and *Ngn3* mRNA expression. Furthermore, treatment of explant cultures with Ang-(1-7) increased mRNA levels of *Insulin* and pancreatic progenitor marker *Ngn3*, as well as *Nox4*, the ROS generation enzyme; these stimulatory effects were attenuated by co-treatment with A779, suggesting that Ang-(1-7), via Mas receptor signaling, may promote differentiation of pancreatic progenitors into insulin-producing cells via modulation of reactive oxygen species. These data together suggest that a Mas receptor-mediated mechanism may stimulate pancreatic cell development.

## Introduction

There is very limited supply of transplantable islet cells and this circumstance impedes the advancement of islet transplantation as a curative treatment for Type 1 diabetes, a chronic disease resulting from the body's failure to produce insulin. Therefore, there is a need for new therapeutic strategies that can promote *in vivo* neogenesis of pancreatic islets and their component cells, especially β-cells, or other cells with β cell-like functions. Elucidation of the developmental biology of the endocrine pancreas is a necessity for the development of therapeutic β-cell regeneration, an alternative therapeutic approach to curing diabetes.

Early expression of renin-angiotensin system (RAS) components in various fetal tissues, such as heart, lung, and kidney [1,2,3], and the existence of local RAS components in the pancreas [4] have led us to investigate local RAS involvement in pancreatic islet function and structure during embryonic development. Angiotensin-(1–7) [Ang-(1–7)] is formed from angiotensin II (Ang II) by angiotensin-converting enzyme 2 (ACE2) [5]. The effects of Ang-(1–7) are mediated through its G-protein coupled receptor, Mas, which is expressed in several tissues, including the heart, kidney and ovary [6,7]. This newly identified ACE2/Ang-(1–7)/Mas axis, which is distinct from the classical RAS pathway, is gaining research importance and has been suggested to act as a negative regulator of Ang II signaling, especially in the attenuation of cardiovascular dysfunction and associated metabolic diseases including diabetes [8,9,10,11].

In diabetic animal models, we [12] and others [8,13,14] have demonstrated that the ACE2/Ang-(1–7)/Mas axis plays a beneficial role in attenuating the development of diabetes in association with islet damage; and that it’s activation is associated with improved insulin sensitivity, pancreatic blood flow, and glucose uptake, underscoring the potential of this axis as a therapeutic target for diabetic treatment. Its presence during organ development has not yet been examined thoroughly. Briefly, it has been shown that ACE2 is abundant in early-gestation placenta and localized to the syncytiotrophoblasts, where it is can regulate the release of Ang-(1–7) into maternal circulation and contribute to vasodilation of the maternal vasculature [15]. ACE2 knockout mice exhibit reduced weight gain and plasma Ang-(1–7) levels during pregnancy [16]. Infusion of Ang-(1–7) into the kidney of ovine fetuses elevated mRNA expression of other RAS components and elevated the osmolality of the amniotic fluid, implicating Ang-(1–7) in fetal kidney development [17].

Although the ACE2/Ang-(1–7)/Mas axis has been linked to pancreatic function in diabetic models, little is known about its involvement in organogenesis [7,16]. To the best of our knowledge, no study thus far has examined whether this axis exists *in vivo* during embryonic development, or whether pharmacological manipulation of this axis can affect intrauterine endocrine cell development in pancreatic islets. Therefore, the present study investigated the expression of ACE2/Ang-(1–7)/Mas axis components in the developing pancreas from embryonic day 12.5 (E12.5) onwards, which is defined as the second transition of islet endocrine expansion and differentiation [18], and we also examined how manipulations of Ang-(1–7) affect the development of the pancreas. Specifically, we investigated the effects of prenatal ACE2 inhibition and Mas receptor blockade on neonatal islet cell composition. Because β-cell replication represents an important mechanism contributing to the expansion of the β-cell population during fetal pancreatic development [19], we also investigated whether manipulations of Mas receptor activation affect the proliferation of existing insulin-positive cells and transcription of genes including *Insulin* and *Ngn3*. Finally, because signaling pathways for reactive oxygen species (ROS) have been shown to be involved in differentiation and angiogenesis [20], we assessed how manipulations of Mas receptor activation and NADPH oxidase inhibition affect ROS levels and transcription of genes of interest in pancreatic explant cultures.

## Materials and Methods

### Ethics statement

Timed-pregnant ICR mice were supplied by the Laboratory Animal Services Centre of the Chinese University of Hong Kong (CUHK). The animals, weighing 30–60 g, were maintained with a 12-hr light/dark cycle in a controlled temperature and humidity environment and were fed with standard food and tap water *ad libitum*, except when fasted for glucose tolerance testing. The experimental protocols used in the present study were approved by the Animal Experimentation Ethics Committee of the CUHK (Ref. 14-025-MIS) and the experiments were performed according to institutional guidelines for the care, and use, of animals in scientific research. Animals were terminated by CO_2_ inhalation and every effort was made to minimize any suffering.

### Pregnant mouse model and harvesting of embryos

ICR mouse embryos were harvested at designated time points (E12.5–E18.5 with E0.5 being the day of vaginal plug). Mouse embryos and neonates were dissected in ice-cold phosphate buffered saline (PBS, pH 7.4; Invitrogen, Carlsbad, CA) under a dissection microscope (Leica Microsystems, Wetzlar, Germany). The isolated pancreata were blotted dry and weighed before utilization in functional studies.

### Drugs

The Mas receptor antagonist A779 (Asp-Arg-Val-Tyr-Ile-His-[D-Ala]) and the specific ACE2 inhibitor DX600 (Ac-Gly-Asp-Tyr-Ser-His-Cys-Ser-Pro-Leu-Arg-Tyr-Tyr-Pro-Trp-Trp-Lys-Cys-Thr-Tyr-Pro-Asp-Pro-Glu-Gly-Gly-Gly-NH2), were purchased from Phoenix Pharmaceuticals Inc. (St. Joseph, MO). Ang-(1–7) was purchased from Bachem Americas (Torrance, CA) and diphenyleneiodonium chloride (DPI) was purchased from Sigma (St. Louis, MO).

### Drug treatment of pregnant mice

Pregnant mice were divided into the following three groups (N = 10 per group): Normal controls (no drug treatment), DX600 (10 μg/kg/day), and A779 (10mg/kg/day). Both drug treatments were delivered by daily intraperitoneal injections for 7 days after being dissolved in normal saline (0.9% w/v). Drug dosages were based on previous reports with modification [14,21]. All parameters reported in the Results section were assessed after the neonates were born.

### Islet isolation from neonatal mouse pancreas

Protocols for islet isolation from neonatal mouse pancreata were modified from previously reported protocols developed in our laboratory [22,23]. In brief, the dissected pancreata from five neonatal mice from each treatment group were pooled together for islet isolation. They were minced finely and digested in 3 mg/ml collagenase P (Roche Diagnostic, Indianapolis, IN) in 5 ml of Hanks balanced salts solution (HBSS) (Sigma-Aldrich, St. Louis, MO) for 7–10 min at 37°C with vigorous shaking. The digestion was terminated by addition of ice-cold HBSS followed by centrifugation for 5 min at 1600 rpm twice, and pancreatic islet tissues were then separated by Histopaque-1077 (Sigma-Aldrich) gradient density centrifugation. The re-suspended islets were handpicked under a stereomicroscope and then cultured in RPMI1640 culture medium supplemented with 10% fetal bovine serum, 1% penicillin, and streptomycin (Invitrogen, Carlsbad, CA) for 3 days before being subjected to functional assessment.

### Glucose-stimulated insulin secretion (GSIS)

GSIS measurements from pancreatic islets were evaluated as described in detail previously [22,23]. Approximately 20 neonatal islets were collected from each group and pre-incubated in Krebs-Ringer bicarbonate buffer (KRBB) containing 1.6 mM D-glucose (Sigma-Aldrich) for 1h to normalize the basal insulin-secreting status before being incubated in KRBB buffer with 1.6 mM D-glucose for 1h after which the buffer was collected; the islets were then incubated for an additional hour with fresh KRBB buffer containing 16.7 mM D-glucose to determine the stimulated level of insulin secretion. Insulin release was quantitated by subjecting collected buffer samples for enzyme-linked immunosorbent assays (ELISAs) with a specific mouse insulin ELISA kit (Antibody and Immunoassay Services at the University of Hong Kong). Insulin secretion rates were calculated as the quantity of insulin accumulated in the supernatant buffer, in an hour, divided by the number of islets (ng insulin per islet per hour)

### Intraperitoneal glucose tolerance test (IPGTT)

Glucose tolerance was tested in 3–4 weeks old pups with an IPGTT as described previously [23] with minor modifications. Briefly, the pups were fasted for 16 h before being given intraperitoneal injections of glucose dissolved in saline (1 g/kg body weight). Blood glucose levels were assessed by collecting blood from a tail tip snip at five time points (0 min, 15 min, 30 min, 60 min, and 120 min after glucose administration). Blood glucose levels were measured with a handheld glucometer (Bayer Corporation, Emeryville, CA).

### Fluorescent immunohistochemistry (IHC)

Neonatal pancreata, islet cultures, and embryonic pancreata were embedded in OCT medium (Sakura Finetek, Torrance, CA), and immediately snap-frozen on dry-ice; and the tissue blocks were then cryosectioned (5 μm) onto Superfrost plus slides in a Leica CM1100 Benchtop Cryostat (Leica Microsystems, Wetzlar, Germany). The slides were first fixed with 4% (wt/vol) paraformaldehyde (Sigma-Aldrich, St. Louis, MO) in PBS for 30 min at room temperature and then permeabilized with a solution containing 0.1% Triton X-100 in PBS before being blocked with 2% BSA (Sigma, St. Louis, MO) at room temperature for 1 h before being incubated with primary antibodies (as detailed in Table 1) at 4°C overnight. Following three washes with PBS, the slides carrying the sections were incubated for 1 h at room temperature with fluorophore-labeled secondary antibodies (Alexa Fluor 488 goat anti-mouse IgG, A11011, 1:200; donkey anti-goat, A11055, 1:400; anti-guinea pig, A11073, 1:400; Alexa fluor 568 goat anti-mouse, A11004, 1:200; and donkey anti-rabbit, A10042, 1:200; Invitrogen). In some experiments, 4’6’-diamidino-2-phenylindole (DAPI, D3571, 1:1000, Invitrogen) was applied after the secondary antibody to counterstain the nuclei. After three washes with PBS, the slides were mounted with Vectashield medium (Vector Laboratories Inc., Burlingame, CA). In all cases, omission of primary antibodies was used as a negative control for detection of non-specific antibody binding. Imaging was done with by fluorescent/light microscopy, using a built in DC200 digital camera system (Leica Microsystems).

**Table 1:** Primary antibodies used for immunofluorescence staining

Name	Catalogue Number	Manufacturer	Dilution
Rabbit anti-ACE2	NBP1-76614	Novus Biologicals	1:500
Rabbit anti-Mas1	NBP1-60091	Novus Biologicals	1:500
Mouse anti-Ngn3	ab54743	Abcam, Cambridge	1:100
Rabbit anti-pdx-1	ab3503	Millipore, Temecula	1:200
Guinea-pig anti-insulin	18–0067	Invitrogen	1:100
Rabbit anti-insulin	sc-9168	Santa Cruz Biotechnology	1:300
Mouse anti-glucagon	ab10988	Abcam, Cambridge	1:250
Rabbit anti-Ki67	ab15580	Abcam, Cambridge	1:500

Islet β-cell area was assessed by determining the proportion of area occupied by Alexa Fluor 488 or 568 labeling within each islet using Leica Qwin image analysis software (Leica Microsystems), as described previously [22,23]. At least five islets from each experimental group and islets isolated from at least five different mother mice were chosen randomly for analysis. All data were expressed as means ± SEMs.

### Histology

Paraffin-embedded slides of neonatal pancreatic sections were deparaffinized in xylene and rehydrated through an ethanol gradient (100%, 95%, 80%, and 70%). The rehydrated slides were rinsed in water and then stained with hematoxylin for 1–2 min to allow visualization of cell nuclei. Any undesirable overstaining was removed by quick-dip in 0.3% acid ethanol, followed by neutralization in Scott’s tap water. Cellular cytoplasm was then counterstained with 1% eosin for 5 min. The slides were then rinsed in water, followed by dehydration through an ethanol gradient (70%, 80%, 95%, and 100%) and cleared with two 1-min dips in xylene. Finally, the haematoxylin and eosin stained (H&E) slides were mounted with DPX rapid-mounting media (WVR International, Lutterworth, UK) and examined by light microscopy (Leica Microsystems).

### Dihydroethidium (DHE) staining and image analysis

Intracellular levels of ROS were detected using DHE, a reduced form of the DNA dye ethidium bromide. DHE was dissolved in DMSO (stock solution, 30 mM) and then diluted in PBS to a 30 μM working solution. Freshly dissected pancreatic rudiments were embedded in O.C.T compound and frozen immediately in liquid nitrogen. Five-micron-thick sections were collected onto glass slides and the slides were then incubated in freshly prepared DHE solution (Sigma-Aldrich, St. Louis, MO) for 30 min in a dark chamber at room temperature, counterstained with DAPI (Invitrogen) for 5 min, and then mounted with Vectashield (Vector Laboratories Inc., Burlingame, CA).

Digital images were acquired via a fluorescence microscope equipped with a DC200 digital camera (Leica Microsystems). The intensity of fluorescence in the collected images was analyzed by Leica Qwin image analysis software (Leica Microsystems). For each group, 9–12 images were collected from three tissue blocks, each of which contained 6–8 embryos.

### Enzyme immunoassay (EIA) of Ang-(1–7) production

Sample embryonic pancreata were collected for each day of gestation from E12.5 onward. Proteins were extracted with an Ang-(1–7) EIA kit (Bachem Americas, Torrance, CA) based on an Ang-(1–7) sequence of H-Asp-Arg-Val-Tyr-Ile-His-Pro-OH.

### Organ explant culture

Explant cultures were produced as described in our previous work [23] with minor modifications. Briefly, E13.5 embryos were harvested and their pancreata were isolated in HBSS (Sigma-Aldrich, St. Louis, MO). The pancreas explants were transferred to culture plate inserts (Millipore, Billerica, MA) in sterile 6-well plates containing RPMI1640 medium (Invitrogen) supplemented with 10% fetal bovine serum, 1% penicillin and streptomycin, 10mM HEPES buffer, and 1x non-essential amino acids (Invitrogen). Group-specified treatments were applied at 37°C in a 5% CO_2_/95% humidified atmosphere for 3–5 days and the cultures were provided with fresh incubation medium every day.

### Real-time reverse transcriptase-polymerase chain reaction (RT-PCR) analysis

Total RNA was extracted from homogenized pancreas tissues, cultured explants, and isolated islet cultures with TRIzol reagent (Invitrogen), according to the manufacturer’s instructions. cDNAs were prepared with a PrimeScript reverse transcriptase master mix kit (Takara Bio Inc., Shiga, Japan). The cDNA templates were combined with SYBRgreen QPCR master mix (Applied Biosystems, Carlsbad, CA) and subjected, in triplicate, to quantitative StepOnePlus Real-Time PCR System (Applied Biosystems, Carlsbad, CA) under standard conditions. The results were normalized to β-actin or GAPDH, and expressed as relative mRNA expression levels using the 2^-ΔΔCT^ statistical method (Desgraz and Herrara, 2009). Melting curve analysis was performed to confirm amplification specificity of the products. The sequences of the primers (Invitrogen, Hong Kong) used are reported in Table 2.

**Table 2:** Gene-specific mouse primers for real-time PCR.

Name of Genes	Forward primers	Reverse primers
Mouse ACE2	TGTGTCAGAAATGTGCGCTTC	CAAGGCGTATCTGTCACAGTC
Mouse Mas1	CTCTCCACCTCCTGACTGTTG	CTGGCAGGATCACAGAGTTTC
Mouse Ngn3	TGCAGCCACATCAAACTCTC	GGTCACCCTGGAAAAAGTGA
Mouse Insulin	CTGGTGCAGCACTGATCTACA	AGCGTGGCTTCTTCTACACAC
Mouse Pdx-1	GAAATCCACCAAAGCTCACG	TTCAACATCACTGCCAGCTC
Mouse Pax4	CTCGAATTGCCCAGCTAAAG	TTACTGTGGGGACTGGGAAG
Mouse Nkx2.2	CCGAGTGCTCTTCTCCAAAG	CTCCACCTTGCGGACACTAT
Mouse Nkx6.1	ACTTGGCAGGACCAGAGAGA	AGAGTTCGGGTCCAGAGGTT
Mouse Glut2	GTCCAGAAAGCCCCAGATACC	GTGACATCCTCAGTTCCTCTTAG
Mouse NOX1	AGGTCGTGATTACCAAGGTTGTC	AAGCCTCGCTTCCTCATCTG
Mouse NOX2	AGCTATGAGGTGGTGATGTTAGTGG	CACAATATTTGTACCAGACAGACTTGAG
Mouse NOX4	CCCAAGTTCCAAGCTCATTTCC	TGGTGACAGGTTTGTTGCTCCT
Mouse p22^phox^	GGAGCGATGTGGACAGAAGTA	GGTTTAGGCTCAATGGGAGTC
Mouse GAPDH	TGGCAAAGTGGAGATTGTTGCC	AAGATGGTGATGGGCTTCCCG

### Western blot analysis

The abundance of ACE2, Mas, and insulin proteins was examined by western blot. Pancreas specimens were homogenized in RIPA buffer (Thermo Scientific, Rockford, IL) and total protein was extracted from the homogenates. Protein concentration was measured using a protein assay kit (Bio-Rad, Hercules, CA) and then adjusted by addition of 2 × SDS sample buffer to equal concentrations across the samples. The adjusted samples were loaded into a 10% SDS-PAGE gel (20 μg protein/well) and subjected to electrophoresis. The fractionized-protein gel was transferred electrically to a PVDF membrane, which was first incubated with blocking solution (5% skim milk in PBS) for 1 h at room temperature to block nonspecific binding, and then probed with primary antibody (anti-ACE2 [NBP1-76614, 1:1000, Novus biologicals], anti-Mas1 receptor [NBP1-60091, 1:1000, Novus Biologicals], anti-p22^phox^ [sc-20781, 1:1000, Santa Cruz Biotechnology], and mouse monoclonal anti-β-actin IgG [sc-47778, 1:1500, Santa Cruz Biotechnology]; all primary antibodies are rabbit polyclonal unless otherwise stated). The membrane was then washed in PBS with 0.1% Tween 20 and probed with peroxidase-labeled anti-rabbit IgG (NA934V, 1:1500, Amersham) or anti-mouse IgG (NA931V, 1:2000, Amersham) secondary antibodies for 1 h at room temperature. The target gene bands were developed with chemiluminescence ECL detection reagent (Amersham, Buckinghamshire, UK), using autoradiography film (Fujifilm, Tokyo, Japan). Band intensities were normalized to β-actin.

### Statistical data analysis

Data are expressed as means ± SEMs. Multiple comparisons between groups were performed using one-way or two-way analyses of variance (ANOVAs) followed by Tukey’s *post hoc* tests as appropriate. Graphics and statistical analysis were produced using GraphPad Prism 5 (GraphPad Software, San Diego, CA). Values of *p* < 0.05 were considered statistically significant.

## Results

### Expression of ACE2/Ang-(1–7)/Mas axis components in embryonic mouse pancreas

**Fig 1:**
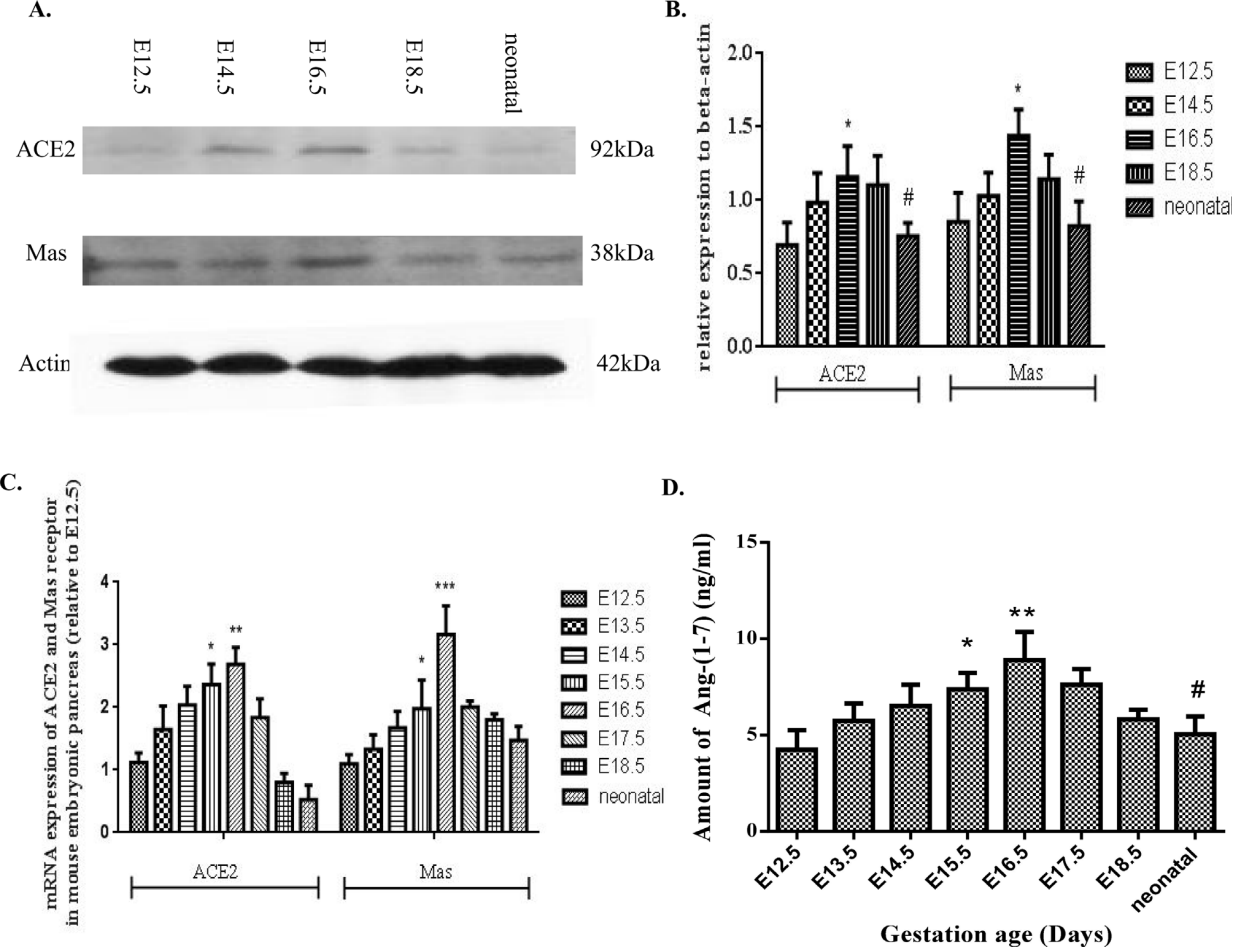
Expression of ACE2 and the Mas receptor in embryonic mouse pancreas from E12.5 to neonatal period. (A-B) ACE2 and Mas receptor protein expression levels in relation to E12.5 levels. n = 6 per time point. (C) Relative mRNA expression of ACE2 and Mas receptor in the developing pancreas. n = 4 per time point. (D) Quantification of Ang-(1–7) levels in the developing pancreas. n = 4 per time point. One-way ANOVA followed by Tukey’s *post hoc* tests were used. All data are expressed as means ± SEM. *p < 0.05, **p < 0.01, ***p < 0.001 *vs*. E12.5, ^#^ *p* < 0.05 *vs*. E16.5.

Western blot and real-time PCR studies have shown consistent protein and mRNA expression of both ACE2 and Mas receptor in embryonic mouse pancreas (Fig 1A–1C), with peak mRNA expression and protein level occurring on E16.5 in both cases. Expression of Ang-(1–7) was also assessed throughout gestation, and, in line with findings for the expression of ACE2 and Mas receptor, the peak for Ang-(1–7) content was also seen at E16.5 (9.381±0.3956 ng/ml) which is significant higher than that observed at E12.5 (3.295±0.9037 ng/ml) (Fig 1D).

**Fig 2:**
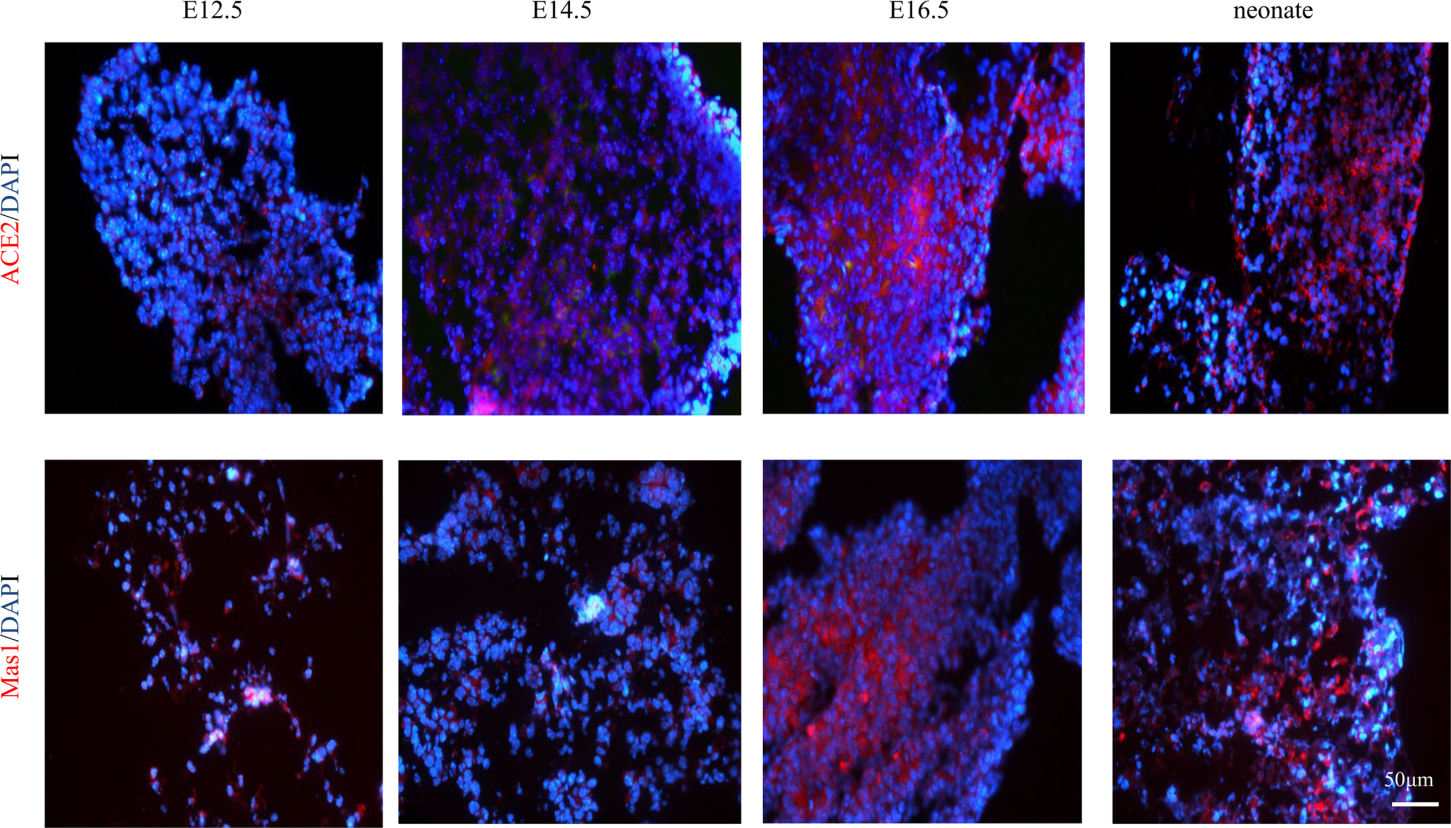
Localization of ACE2 and the Mas receptor at E12.5, E14.5, E16.5, in embryonic mouse pancreata. Florescent IHC for ACE2 and the Mas receptor are shown above and below, respectively (both are shown in red). Both series were nuclear counterstained with DAPI (blue). Scale bar = 50 μm.

As shown in Fig 2, fluorescent IHC confirmed the presence of ACE2 and Mas receptor proteins throughout the gestation period studied (E12.5 to E18.5). Both proteins were similarly localized in the cytoplasm of the neonatal pancreas (Fig 2). Examination of the histology over this developmental period also revealed the formation of primitive islet clusters through a process of major differentiation and maturation of endocrine cells.

### Effects of ACE2 inhibition and Mas receptor antagonism *in utero* on neonatal pancreatic endocrine cell composition

**Fig 3:**
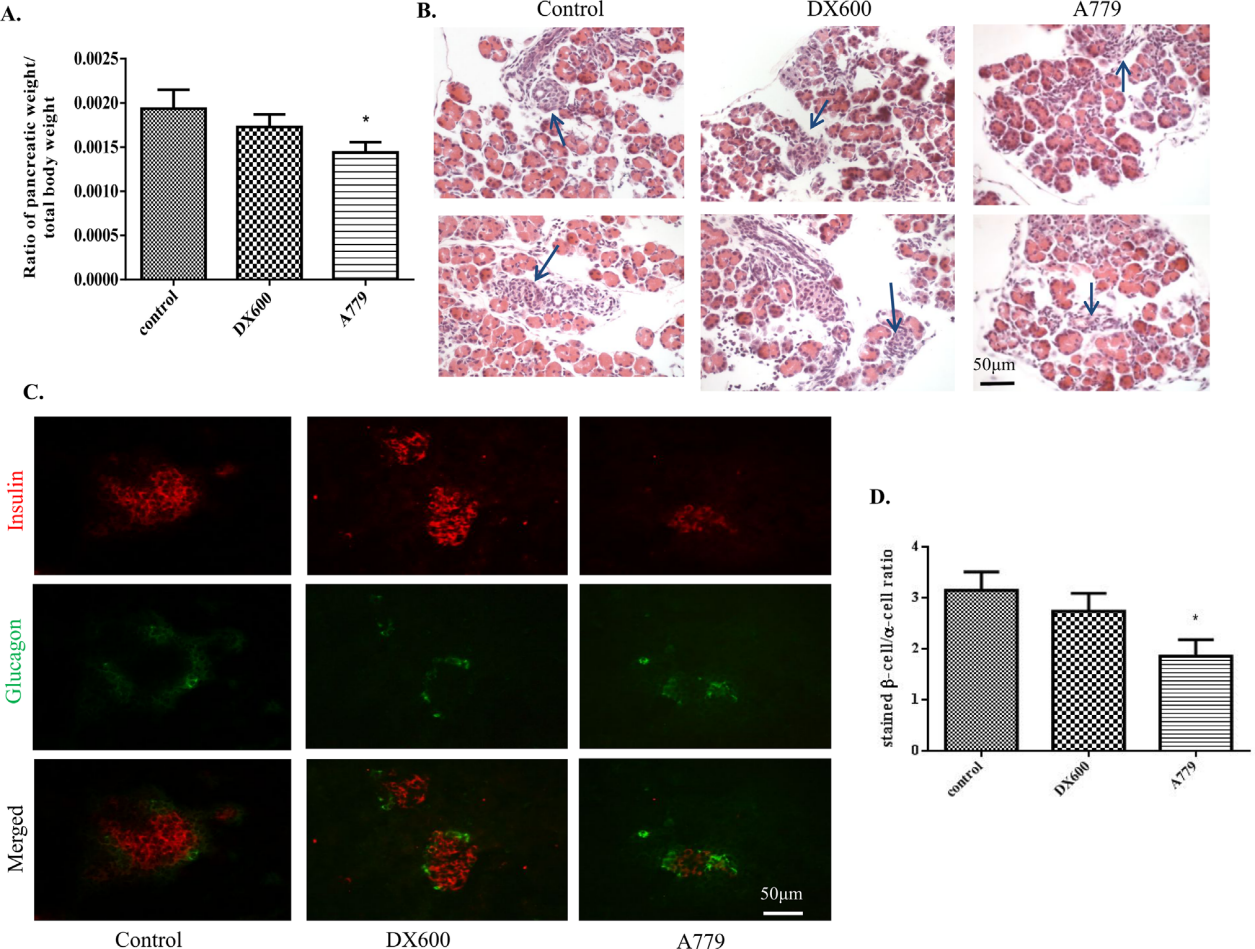
Effects of ACE2 inhibition and Mas receptor antagonism on endocrine cell composition of neonatal mouse pancreas (day 4 postnatal). (A) Ratio of pancreas weight to total body weight of neonates. n = 7 and each group included 3 neonatal mice each time. One-way ANOVA followed by Tukey’s *post hoc* tests were performed. (B) H&E sections showing altered structure (blue arrows) of pancreata from pups in the A779 (Mas receptor antagonist) group, but not the DX600 (ACE2 inhibitor) group, compared to controls. Scale bar = 50 μm. (C-D) Assessment of β-cell and α-cell areas based on fluorescent IHC through insulin and glucagon staining. At least six islets were examined from each animal and at least 5 mice were employed in each group. One-way ANOVA followed by Tukey’s *post hoc* tests were performed. All data are expressed as means ± SEM. *p < 0.05 *vs*. control.

Neither the specific ACE2 inhibitor DX600 nor the Mas receptor antagonist A779 treatment significantly affected blood glucose levels or body weight of the treated pregnant mice (Tables 3 and 4), even with the trend that A779 has increased blood glucose levels as compared to control (Table 4). As shown in Fig 3A, the ratio of pancreatic weight to total body weight in the neonatal pups of the A779-treated group was significantly reduced compared to that of control group pups (0.001514 ± 0.0001265 *vs*. 0.001924 ± 0.0003673, *p* < 0.05), but no such effect was seen in the DX600 group pups (0.001602 ± 0.000288) (Fig 3A). Furthermore, H&E-stained neonatal pancreas sections from the A779 group pups showed structurally disrupted islets, whereas the islets from the DX600 group pups appeared to be histologically intact and comparable to control pup islets (Fig 3B).

**Table 3:** Body weight of the neonatal pups from whose mom is treated with DX600 and A779 during pregnancy.

Groups	Days				
	2	4	6	8	10
**Control**	1.53±0.03	2.20±0.06	3.43±0.09	5.27±0.70	6.64±0.16
**DX600**	1.57±0.02	2.05±0.04	3.66±0.04	5.50±0.07	6.90±0.06
**A779**	1.56±0.02	2.27±0.03	3.93±0.04	5.83±0.06	7.38±0.06

Body weight (g):

**Table 4:** Blood glucose levels of the neonatal pups from whose mom is treated with DX600 and A779 during pregnancy.

Group	Days							
	2	4	6	8	10	12	15	18
**Control**	4.97±0.18	5.54±0.24	5.74±0.36	6.21±0.24	6.77±0.27	6.75±0.24	7.11±0.37	7.16±0.35
**DX600**	5.16±0.21	5.97±0.13	6.39±0.19	6.49±0.09	6.49±0.25	7.59±0.23	8.12±0.39	7.91±0.31
**A779**	5.11±0.18	5.77±0.13	5.83±0.22	5.96±0.25	7.28±0.11	8.23±0.15	8.49±0.66	7.96±0.51

Blood Glucose (mmol/L):

As shown in Fig 3C, fluorescent IHC for localization of insulin and glucagon in neonatal islets revealed a trend toward a decrease of β-cell area per islet and a significantly lower β-cell/α-cell ratio in isolated islets from the A779 group versus the values obtained for control group (Fig 3C and 3D). These changes in the A779 group were accompanied by a shift of some α-cells toward the islet centers. Thus, attenuation of Mas receptor treated by A779 resulted in abnormal differentiation of endocrine cells.

### Mas receptor antagonism *in utero* alters serum insulin secretion, glucose tolerance, and endocrine cell differentiation in neonatal mice

**Fig 4:**
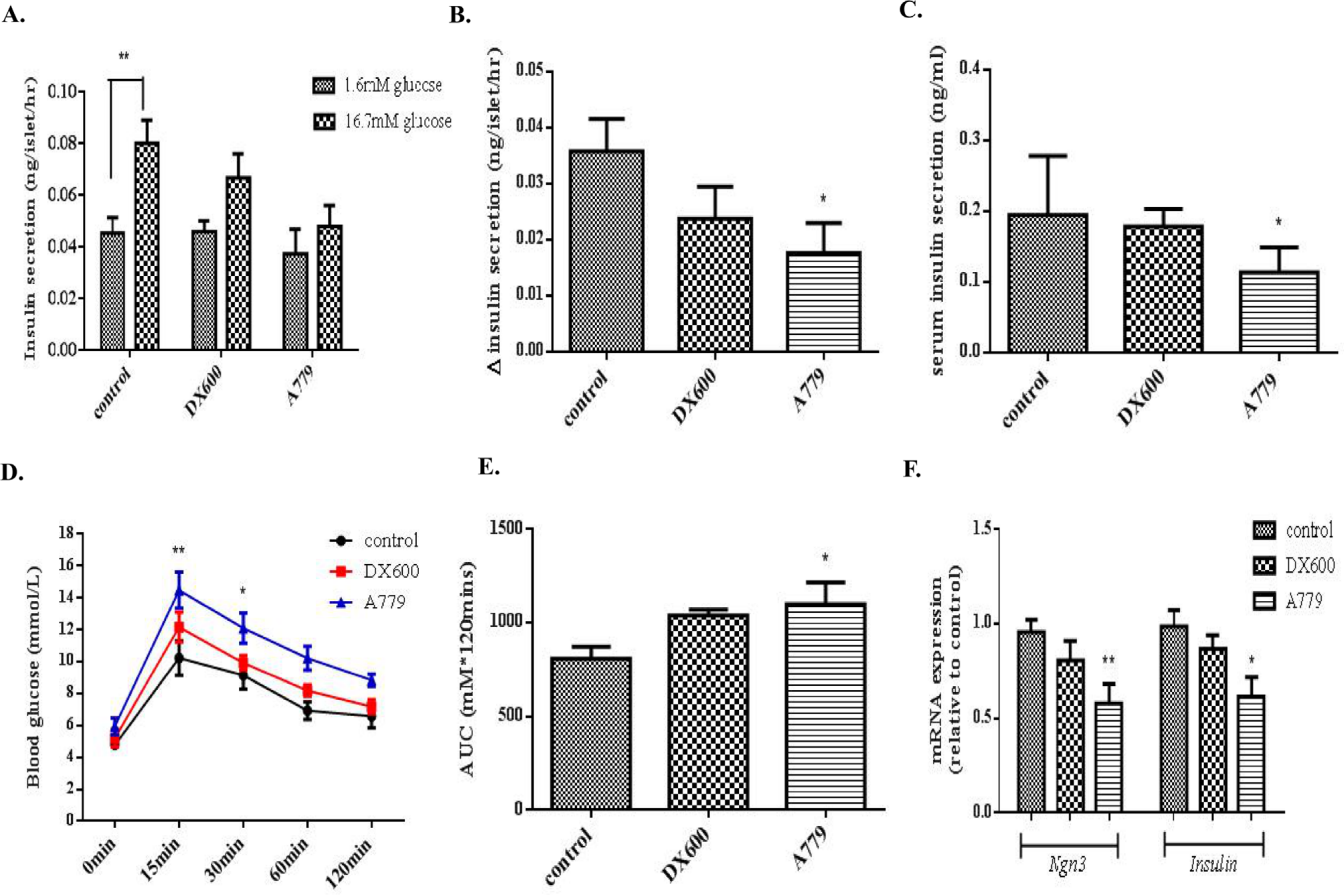
Insulin secretory function and glucose tolerance in mouse neonates. (A-B) Insulin release from neonatal islets (day 4 postnatal) in response to a high-glucose (16.7 mM) challenge. One-way ANOVA followed by Tukey’s *post hoc* tests were used. (C) Serum insulin concentration in serum collected from neonates (day 4) from each group. One-way ANOVA followed by Tukey’s *post hoc* tests were performed. (D) IPGTTs were performed on 4-weeks-old pups from each experimental group. Changes in blood glucose level were measured immediately before (t = 0 min), and at four time points after (t = 15, 30, 60 and 120 min), glucose loading. Two-way ANOVA followed by Tukey’s *post hoc* tests were used. (E) Comparison of areas under the curve (AUCs) across groups. (F) mRNA expression of *Ngn3* and *Insulin* in neonatal islets isolated from DX600 or A779 group pups. One-way ANOVA followed by Tukey’s *post hoc* tests were performed. All data are expressed as means ± SEM, n = 6 per group. *p < 0.05, **p < 0.01 *vs*. control.

Islets isolated from control neonatal pups exhibited normal up-regulation of insulin release in response to a high-glucose challenge (Fig 4A), whereas a severely reduced GSIS was observed in islets from A779 group pups (Fig 4B). The A779 group pups also had significant decreased serum insulin content compared to controls (0.111 ± 0.01451 ng/ml *vs*. 0.1945 ± 0.02472 ng/ml; *p* < 0.05) (Fig 4C).

Basal blood glucose levels after 16 h of fasting did not differ between the A779 and control groups for one-month old mice, but the A779 group had significantly elevated peak glucose levels during IPGTTs, relative to controls, most notably at the 15-min and 30-min time points (Fig 4D). Analysis of the areas under the curve (AUCs) revealed that the A779 group pups had reduced glucose tolerance relative to control group pups (1099 ± 116 *vs*. 807.6 ± 64.1, *p* < 0.05) (Fig 4E). In addition, real time-PCR analysis revealed a significant reduction in both *Ngn3* and *Insulin* mRNA transcription expression in islets from neonatal pups in the A779 group relative to expression levels observed in islets from control group pups (Fig 4F).

### Exogenous Ang-(1–7) alters mRNA expression of endocrine cell markers and differentiation of insulin-synthesizing cells in embryonic pancreas explants

**Fig 5:**
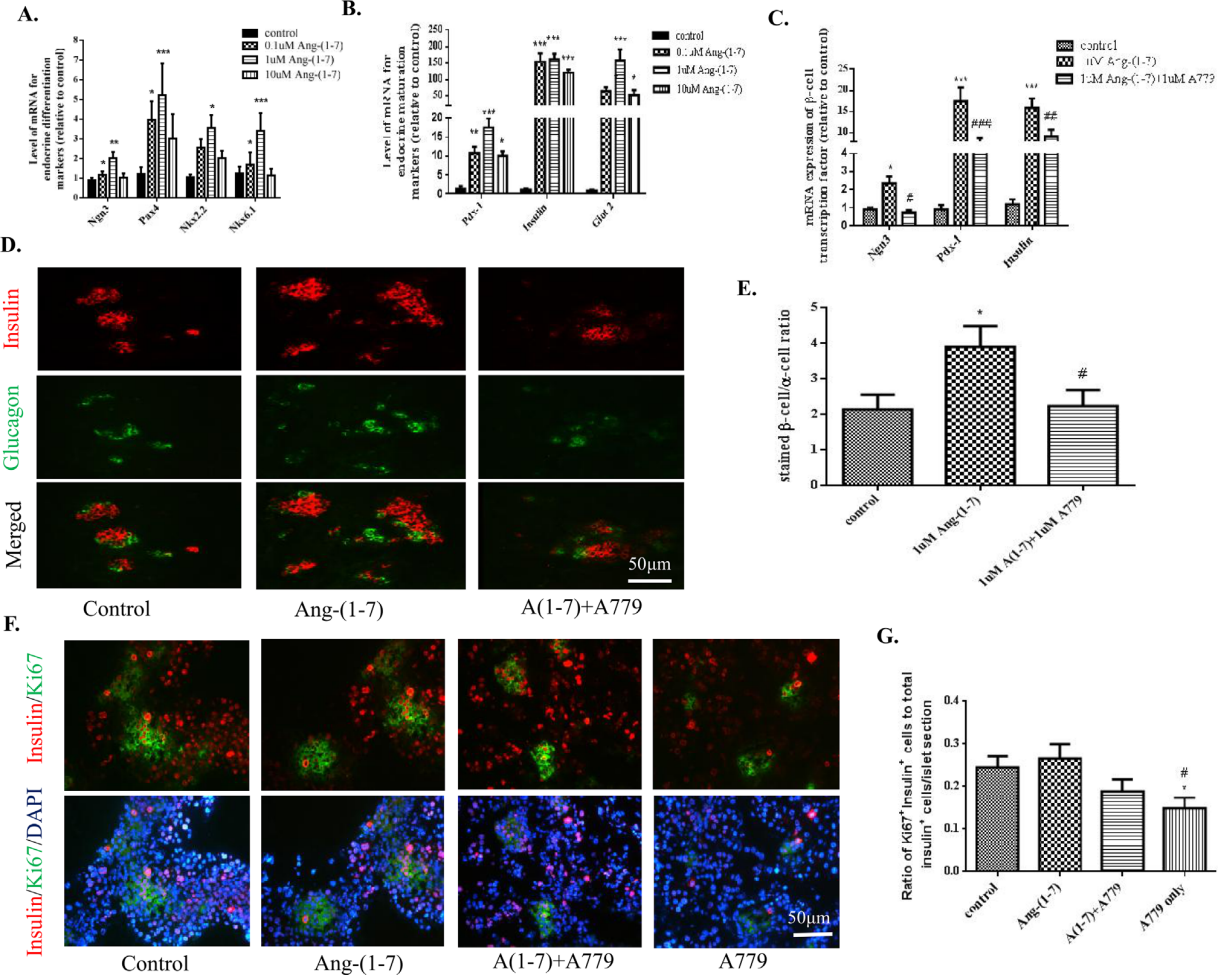
Effects of Ang-(1–7) and A779 on endocrine cell differentiation in cultured pancreatic rudiments. (A-B) Expression of endocrine cell marker transcripts following administration of Ang-(1–7) at a range of doses. (C) Real-time assessment of the mRNA expression of *Ngn3* and *Insulin*. n = 6. (D-E) Assessment of β-cell and α-cell areas as identified by immunofluorescent staining with anti-insulin and anti-glucagon antibodies. (F-G) Quantitation of proliferating β-cells, recognized as insulin^+^Ki67^+^cells, in pancreas explant cultures exposed to exogenous 1 μM Ang-(1–7) or 1 μM A779. Scale bar = 50 μm. One-way ANOVA followed by Tukey’s *post hoc* tests were used. All data are expressed as means ± SEMs. n = 6 from at least 7 pancreatic rudiments were used in each group. *p < 0.05, **p < 0.01, *** *p* <0.001 *vs*. control, ^#^ *p* < 0.05, ^##^ *p* < 0.01, ^###^ *p* <0.001 *vs*. Ang-(1–7).

Ang-(1–7) is a peptide, which can be easily degraded by *in vivo* administration; therefore *ex vivo* experiments were performed in explant cultures of embryonic pancreas to examine the effect of Mas receptor activation by Ang-(1–7) on embryonic pancreas development. When *ex vivo* embryonic pancreas explants were exposed to the Mas receptor ligand Ang-(1–7) for 7 days, up-regulation of the mRNA expression of endocrine cell differentiation and maturation markers, including *Ngn3* and *Insulin*, was observed (Fig 5A and 5B), with the most marked effects being observed with the 1 μM dose of Ang-(1–7). The upregulated expression levels of the *Ngn3*, *Pdx-1*, and *Insulin* transcripts were attenuated in the presence of 1 μM A779 (Fig 5C). The *Insulin* mRNA changes were consistent with our fluorescent IHC findings (Fig 5D and 5E). Additionally, the explants treated with A779 alone, but not those treated with Ang-(1–7), had reduced numbers of double-labeled insulin^+^/Ki67^+^ cells compared to untreated explants (Fig 5F and 5G).

### Mas receptor activation augments oxidative stress in embryonic pancreas explants

**Fig 6:**
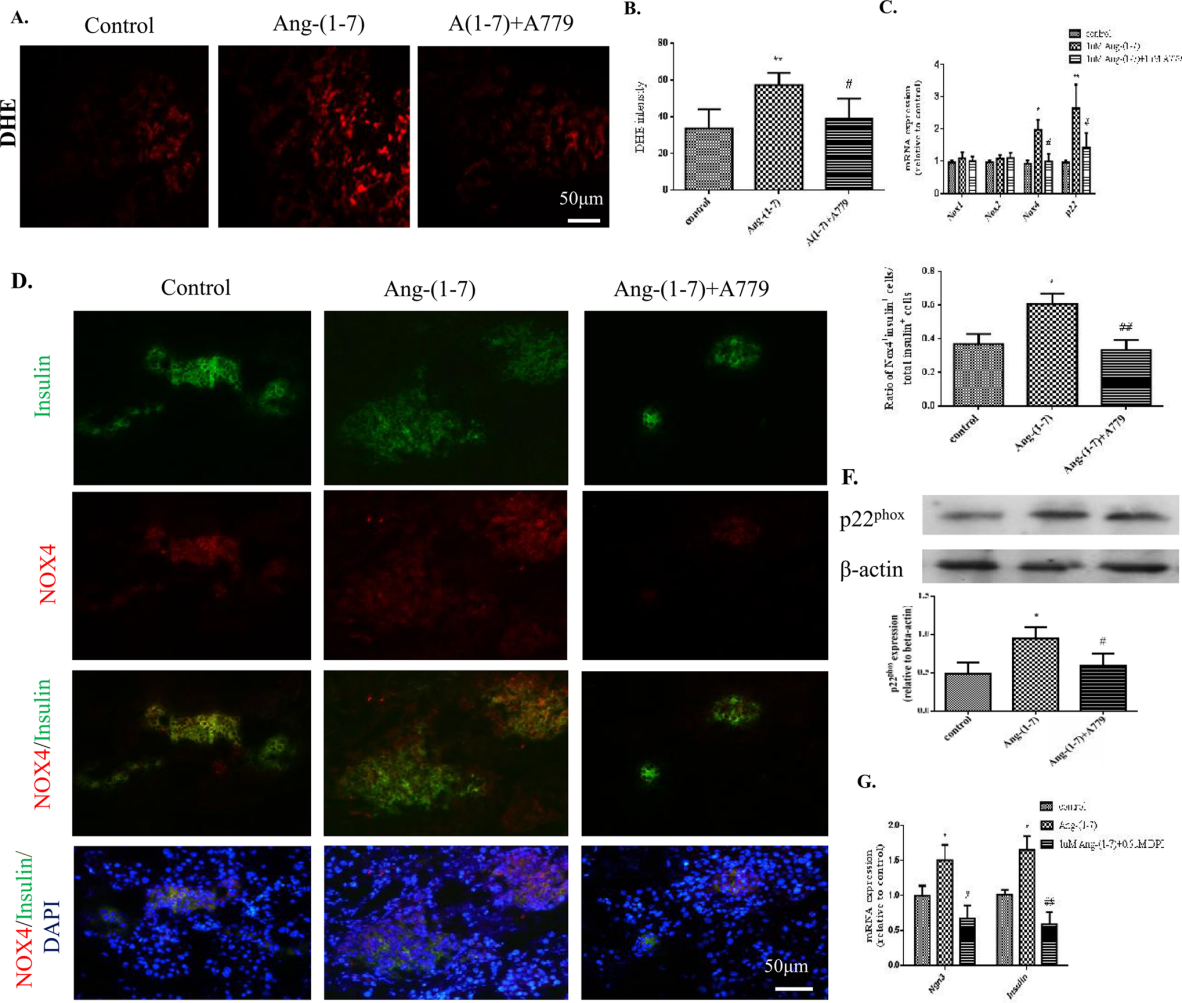
Assessment of the interaction of Ang-(1–7) and ROS in cultured pancreatic rudiments. (A-B) DHE-labelled sections. (C-D) Assessment of mRNA expression changes of NADPH oxidase subunits *Nox1*, *Nox2* and *Nox4*. (E-F) IHC for Nox4. (G) Protein expression of p22^phox^ in pancreatic rudiments treated with 1μM A779 (Mas receptor antagonist) in the presence and absence of 1 μM Ang-(1–7). (H) mRNA expression of *Ngn3* and *Insulin* in pancreatic rudiments treated with 1 μM Ang-(1–7) in the presence and absence of 0.5 μM DPI (Nox4 inhibitor). Scale bar = 50 μm. One-way ANOVA followed by Tukey’s *post hoc* tests were used. All data are expressed as means ± SEM. n = 5 per group. *p < 0.05, **p < 0.01, *** *p* < 0.001 *vs*. control, ^#^ *p* < 0.05, ^##^ *p* <0.01 *vs*. Ang-(1–7).

Ang-(1–7) treatment resulted in increased levels of ROS in pancreatic explant cultures, as shown by DHE labeling, compared to controls, and these elevated ROS levels were attenuated by co-treatment with the Mas receptor antagonist A779 (Fig 6A and 6B). More specifically, real-time RT-PCR experiments revealed that Ang-(1–7) increased transcription of *Nox4*, among other NADPH oxidase family genes, and this effect was also blocked by the addition of A779 (Fig 6C). Fluorescent IHC also demonstrated increased expression of the Nox4 protein in Ang-(1–7)-treated explants and this increase was also decreased significantly in the presence of A779 (Fig 6D and 6E). Likewise, western blots demonstrated that the Ang-(1–7) treatment increased levels of the p22^phox^ protein, a regulatory component for Nox4, and this effect was again blocked by the addition of A779 (Fig 6F). Furthermore, when Ang-(1–7) was added in the presence of 0.5 μM DPI, an NADPH oxidase inhibitor, the increased transcription of *Ngn3* and *Insulin* observed with Ang-(1–7) alone was blocked (Fig 6G).

## Discussion

The present study is the first to present a characterization of the ACE2/Ang-(1–7)/Mas axis in the developing mouse pancreas. Upregulated expression of each component of ACE2/Ang-(1–7)/Mas axis was consistently observed in the developing pancreas throughout embryonic development from E12.5 onward, peaking at E16.5. These observations strongly suggest the implication of ACE2/Ang-(1–7)/Mas axis in the regulation of pancreatic development, at least in mouse embryogenesis. Prenatal treatment with the Mas receptor antagonist A779, but not the ACE2 inhibitor DX600, reduced the β-cell to α-cell ratio of neonatal islets, impaired insulin secretion, and reduced glucose tolerance. Furthermore, A779 also decreased mRNA expression of *Ngn3* (a β-cell progenitor marker) and of *Insulin* (a differentiated β-cell marker) in pancreas explants. Ang-(1–7) increased *Insulin* and *Ngn3* as well as *Nox4* mRNA levels in explants, and these increases were attenuated by A779, suggesting that Ang-(1–7) activation of Mas receptors may promote differentiation of progenitors into insulin-producing cells via ROS modulation.

It is noteworthy that we observed co-localization of ACE2 and Mas receptor positivity with insulin expressing cells in the critical time window for β-cell development (E15.5 onward), supporting the hypothesis that the ACE2 axis plays a regulatory role in endocrine pancreas cell lineage, especially in β-cell maturation (data not shown). Our findings are in keeping with recent studies demonstrating tissue-specific developmental regulation of the expression of ACE2, and other RAS components, in various organs, with increased expression in the late stages of gestation extending into the neonatal period followed by a gradual decrease with maturation [24,25,26]. The postnatal period may be a critical window for islet cell maturation with endocrine cells undergoing additional remodeling and maturation in the 2–3 weeks after birth, in mice [18,27]. ACE2 has been reported to mediate a number of beneficial physiological effects, including relaxation of blood vessels and improvement of heart and renal function via anti-inflammatory and anti-apoptotic effects, and appears to be a negative regulator of RAS [9,10]. In diabetic mice, exogenous ACE2 results in improved glycemic homeostasis, further suggesting an association between ACE2 activity and insulin secretion [13] and re-confirming the beneficial effects of ACE2 in pathophysiological conditions [9,10,28]. The specific ACE2 inhibitor DX600 did not produce any significant effects in the present study, which was performed under conditions of normal (non-pathophysiological) embryonic development. A prior study involving ACE2 knockout mice suggested that ACE2 deficiency was associated with fetal growth restriction [16]. However, in the current study, only an ACE2 blocker was used. Therefore our negative findings with DX600 could be due to ACE2 not being fully blocked systemically. Further studies of specific organ development with ACE2 knockout mice are needed.

The present study provides data suggesting a previously unreported, but major role, of the Mas receptor in pancreatic development and in the maintenance of insulin secretory function in early postnatal life. A few prior studies have investigated the role of placental ACE2 axis activity in organogenesis in animal models [29,30]. In this regard, our histological observations revealed that prenatal Mas receptor antagonism produced severe disruption of islet architecture accompanied by a reduction in total pancreatic mass, suggesting that Mas receptor activation may be key to directing progenitor cells into the β-cell lineage, particularly with respect to determination of whether endocrine progenitor cells adopt an α- or β-cell fate. That is, altered Mas receptor activation may lead to some cellular reprograming, such that some cells become other cell types rather than β-cells, as reported elsewhere [31,32,33]. Importantly, our results showed that blockade of endogenous Mas receptors not only reduced pancreatic development, but also led to reduced islet function up to at least 1 month of age. These results show that inhibition of β-cell development during embryonic/fetal life can have long-term consequences for endocrine pancreas function. Endogenous Mas receptor antagonism in our E12.5 explant culture paradigm reduced levels of both *Ngn3* and *Insulin* mRNA, and resulted in fewer pancreatic progenitor cells differentiating toward the β-cell lineage. Exogenous addition of Ang-(1–7) induced up-regulation of *Ngn3* and *Insulin* mRNA expression. Our finding that the up-regulation of *Ngn3* could be blocked with the addition of the Mas receptor antagonist A779 indicate that this effect, at least for *Ngn3*, is related directly to Ang-(1–7)-mediated Mas receptor activation.

The endocrine pancreas is exceptionally sensitive to variations in intracellular levels of ROS and ROS production depends mainly on the activity of an enzymatic complex-NADPH oxidase, which has been characterized in pancreatic islets and has been implicated in insulin secretion regulation [34,35]. Moreover, increased levels of ROS are often associated with increased stem cell/progenitor cell differentiation [36]. In this context, upregulated ROS levels upon Ang-(1–7) stimulation suggest that Ang-(1–7) may mediate endocrine cell differentiation via ROS modulation. More specifically, our real-time PCR and IHC results indicate that Nox4 and p22^phox^ are present in the developing mouse pancreas and suggest that increased levels of Nox4 have a positive influence on endocrine cell differentiation, a notion that is consistent with the recent discovery that reduced ROS levels lead to reduced β-cell differentiation [37]. Treatment of explants with A779 reversed Ang-(1–7)-mediated changes in the expression of the NADPH oxidase subunit Nox4. Furthermore, in the presence of the NADPH oxidase inhibitor DPI, Ang-(1–7) did not affect differentiation in pancreatic rudiment cultures, providing further evidence for the hypothesis that Ang-(1–7) stimulates the differentiation of pancreatic endocrine cells via a ROS bypass. To the best of our knowledge, this is the first study to implicate Ang-(1–7) as a mediator ROS production. Previously, Ang-(1–7) has been viewed primarily as serving a protective role in various tissues such as lung [38], brain [39,40] and adipose [41] based on data showing that it is associated with inhibition of ROS formation, particularly ROS induced by Ang II [40,42]. Previously, the effects of Ang-(1–7) on Ang II-induced ROS have been studied mainly in the cardiovascular and renal systems; the present study demonstrated that Ang-(1–7) also has a notable effect on ROS mediation in the endocrine pancreas and that this effect is neutralized by A779. Given that Ang II receptors are also present in the embryonic pancreas [23], it remains to be determined if Ang II itself is present in the developing endocrine pancreas and whether local Ang II generation affects Ang-(1–7) mediated-ROS activation. ROS-induced cell development is thought to be mediated by mitogen-activated protein kinase (MAPK) activity, because differentiation can be abolished by manipulations of MAPK [37,42]. Indeed, we did observe reduced phosphorylation of MAPK (specifically, in extracellular signal-regulated kinase-1/2) in neonatal pancreata isolated from pups in the A779 treatment group (data not shown). This finding provides further evidence that Ang-(1–7), acting through Mas receptors, may induce differentiation of pancreatic cell development. More specifically, our findings suggest that this mechanism may involve Ang-(1–7)-mediated ROS generation that leads to MAPK activation.

In conclusion, we have reported, for the first time, on a previously unidentified role of the ACE2/Ang-(1–7)/Mas axis in the regulation of the development of β-cells in mouse embryos *in vivo*. These findings have potential implications for the development of therapies designed to enhance β-cell neogenesis in prediabetic and diabetic patients. Given that decreases in ACE2 axis components have been observed with the progression of diabetes [13,43], reversal or restoration of components of the ACE2 axis by way of *in vivo* cell reprogramming could become a major strategy for restoring β-cell mass, and could provide a valuable tool for reducing morbidity in diabetic patients with inadequate β-cell function.
